# Signalling by potassium: another second messenger to add to the list?

**DOI:** 10.1093/jxb/erx238

**Published:** 2017-07-28

**Authors:** Sergey Shabala

**Affiliations:** 1 Department of Horticulture, Foshan University, Foshan, China; 2 School of Land and Food, University of Tasmania, Private Bag, Hobart, Tas, Australia

**Keywords:** Cytosolic potassium, defence, GORK, homeostasis, metabolism, signalling

**Cytosolic potassium homeostasis and the ability of various tissues to retain potassium under stress have emerged as important for salinity tolerance in plants, but recent evidence suggests that stress-induced K^+^ efflux may be equally important in mediating growth and development under hostile conditions. Here, the evidence is assessed, and the already-proposed concept of potassium efflux being a switch between metabolic and defence responses is developed. A new model is put forward which suggests signalling roles for cytosolic K^+^ changes, alongside well-known cytosolic Ca^2+^ and ROS ‘signatures’.**

Over the past decade, cytosolic potassium homeostasis and the ability of various plant tissues to retain potassium under stress conditions have emerged as novel and essential mechanisms of salinity stress tolerance in plants (reviewed by Shabala and Pottosin, 2014; Shabala *et al.*, 2016*a*). Reported initially for barley roots (Chen *et al*., 2005, 2007*a*,b), a positive correlation between the overall salinity stress tolerance and the ability of root tissue to retain K^+^ was later expanded to other plant species such as wheat (Cuin *et al*., 2008, 2009), lucerne (Smethurst *et al.*, 2008; Guo *et al.*, 2016), pepper (Bojorquez-Quintal *et al.*, 2016), cotton (Wang *et al.*, 2016*b*), cucumber (Redwan *et al.*, 2016), and Arabidopsis (Sun *et al.*, 2015). This trait also explains the inter-specific variability in salinity stress tolerance (poplar – Sun *et al.*, 2009; mangroves – Lu *et al.*, 2013; Brassica – Chakraborty *et al.*, 2016), and has recently emerged as a novel (and essentially overlooked) mechanism of salinity tissue tolerance in shoots (Wu *et al*., 2013, 2015). Differential K^+^ retention ability also confers differential salinity stress tolerance between halophytes and glycophytes (Percey *et al.*, 2016).

Electrophysiological and genetic studies have revealed that K^+^-selective, depolarization-activating outward-rectifying K^+^ channels (GORK channels in Arabidopsis) represent one of the major pathways of salinity-induced K^+^ efflux from plant cells (Demidchik, 2014; Pottosin and Dobrovynskaya, 2014; Shabala *et al.*, 2016*a*). The GORK channel belongs to the so-called Shaker family of transporters. These are multimeric proteins with the trans-membrane core, forming the permeation pathway, composed of four subunits (Very *et al*., 2014). Similar to all K^+^-selective channels, the GORK channel bears a specific signature TxGYG (Thr-X-Gly-Tyr-Gly) in the pore loops (Sharma *et al.*, 2013) that underlies its explicit K^+^ selectivity. The GORK channel is strongly voltage-gated (Very *et al*., 2014) and activated upon membrane depolarization and by reactive oxygen species (ROS) (Demidchik *et al.*, 2010).

The essential nature of the K^+^ retention trait has moved well beyond salinity stress tolerance. For example, the capacity to maintain high cytosolic [K^+^] was shown to be critical for heavy metal tolerance (Murphy and Taiz, 1997). The recent paper from our laboratory has shown that Arabidopsis *gork1-1* mutants lacking functional K^+^ efflux channels possess higher hypoxia stress tolerance (Wang *et al.*, 2016*a*). Earlier, a similar conclusion was reached for oxidative stress tolerance (Demidchik *et al.*, 2010). Thus, the ability of plant tissues to retain K^+^ seems to be a common feature of all stress-tolerant genotypes and species.

## GORK channel puzzles

If potassium retention is so essential for stress tolerance, why do plants have GORK channels? Wouldn’t it be more logical to eliminate them over the course of evolution? Can we ‘assist’ plants in doing this by knocking them out? Will it result in a stress-tolerant phenotype? Before these questions can be answered, GORK functional expression and regulation patterns should be considered at the tissue- and cell-specific level.

To start with, GORK channels are expressed not only in the root epidermis but also in guard cells (hence the name GORK – *G*uard Cell *O*utward *R*ectifying *K*^+^ channels; Very *et al*., 2014) and play an important role in stomatal closure. In shoots, drought stress can cause up-regulation of GORK transcripts (Becker *et al.*, 2003), and disruption of GORK activity resulted in impaired stomatal closure (Hosy *et al.*, 2003) thus compromising the plant’s ability to retain water. Therefore, although having fewer functional GORK channels may help plants reduce K^+^ leakage from the root, the associated yield penalties due to the inability to adjust transpiration to conditions of hyperosmotic stress may override the benefits gained.

Another puzzling piece of information is that plants respond to salinity stress by the *increase* in the GORK transcript level. For example, treating barley roots for 48 h with 100 mM NaCl has resulted in 3.5- to 5-fold increases in the GORK transcript level in barley roots (Adem *et al.*, 2014), regardless of their salinity stress tolerance. In *Brassica* species, this increase was even higher (up to 8-fold in *B. oleracea* and *B. juncea*; Chakraborty *et al.*, 2016). These observations seem to be counter-intuitive. If potassium retention is so crucial, why do plants invest in developing potassium leak pathways? What is the physiological rationale behind this phenomenon?

## Three possible explanations

First, in plant cells K^+^ operates as a charge-balancing ion. When plants are confronted with high salinity, uptake of Na^+^ causes a rapid and massive membrane depolarization (by 60–80 mV; Shabala *et al.*, 2005; Chen *et al.*, 2007*a*). This has major implications for transport of various essential nutrients and metabolites. To restore the membrane potential, plants have two possible options: (1) to increase active H^+^-pumping, or (2) to use K^+^ efflux for charge balance. The second option comes at a lower energy cost and therefore may be preferred, at least in the short term. It should be mentioned in this context that the reported increase in GORK transcript levels in *Brassica* roots was accompanied by a concurrent increase in HAK5 transcript levels (Chakraborty *et al.*, 2016). Interestingly, this increase was highest in salt-tolerant *B. napus* species, consistent with overall higher K^+^ tissue content in this species (Chakraborty *et al.*, 2016) and suggesting a compensation mechanism. Thus, it appears that plants may use K^+^ efflux as a ‘safety valve’ to deal with initial membrane depolarization caused by salinity, until this role is gradually assumed by up-regulated H^+^-ATPases (Wu and Seliskar, 1998; Chen *et al.*, 2007*a*; Alvarez-Pizarro *et al.*, 2009), and then gradually regain K^+^ via high-affinity K^+^ transport systems (HATS).

Potassium is also known as a determinant of cell fate (Shabala, 2009). High cytosolic K^+^ levels are essential for suppression of caspase-like proteases and endonucleases, and Arabidopsis mutants lacking functional GORK channels showed less programmed cell death (PCD) events compared with wild type, under both salinity and oxidative stress conditions (Demidchik *et al.*, 2010). While cell elimination via PCD may generally be considered an undesirable trait, under some circumstances this process may be an essential component of acclimation (e.g. for aerenchyma formation in root cortex under hypoxic conditions; Shabala, 2011).

Finally, K^+^ efflux may represent a ‘metabolic switch’ inhibiting energy-consuming anabolic reactions and saving energy for adaptation and repair. Termed a ‘metabolic hypothesis’ by Demidchik (2014), this concept needs to be proven in direct experiments. The physiological rationale behind it is that K^+^ is known to be an activator of a very large number (>70) of metabolic enzymes (Dreyer and Uozumi, 2011; Anschutz *et al*., 2014). Under control conditions, when cytosolic K^+^ concentrations are high, these enzymes are active and draw the major bulk of available energy towards the metabolic processes driven by these reactions (Box 1). When plants are confronted by stress conditions, they need to redirect a large pool of ATP towards defence reactions, even though ATP production declines dramatically. The only way to achieve this and avoid the competition for energy between metabolic and defence responses is to shut down cell metabolism. Decreasing the cytosolic [K^+^] to sub-threshold levels will inactivate numerous metabolic reactions, allowing a redistribution of the ATP pool towards defence responses (Box 1).

Box 1. **Energy balance and its distribution in stressed plants**

The total pool of available energy is considered as 100% under control conditions and diminishes with increased stress severity (duration). When plant cell cytosolic [K^+^] is high (panel A) the major bulk of the pool is directed towards cell metabolism (70% in the model). As stress progresses, the amount of energy available for defence is quickly reduced to zero, and the cell dies (red area). If the cell uses K^+^ efflux as a metabolic switch and allocates only 30% of initially available energy for metabolism (panel B), cell death occurs much later.

**Figure:**
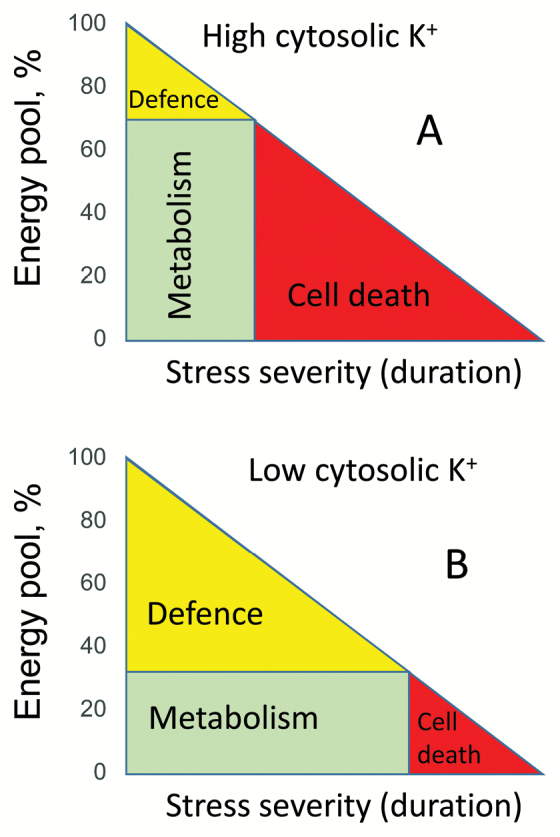

Can plants afford such a mechanism? The answer is yes, assuming the process is tightly controlled (see suggested model in Box 2) and several restrictions are in place. To start with, such rapid K^+^ efflux from the root should be confined to a relatively small root region, thereby ensuring that the overall root potassium nutritional status is not compromised. The root apex seems to be the most likely candidate for such a role (Box 2). First, cells in this zone are very active metabolically and thus best suited for the role of such a switch. Second, these cells show much higher sensitivity to salt, having an overall rate of K^+^ loss 10 to 30 times higher compared with mature zone cells (Shabala *et al.*, 2016*b*). Third, root apical cells have less negative membrane potential (MP) compared with cells in the mature zone, reflecting lower H^+^-ATPase activity in this region (Shabala *et al.*, 2016*b*), and thus should rely more on K^+^ efflux as a means of restoring membrane potential (MP). Fourth, the xylem tissue in this region is underdeveloped so the changes in the radial K^+^ fluxes will have no implications for long-distance K^+^ transport to the shoot. Finally, the overall volume of cells in the apex is much smaller compared with the bulk of the root, made of mature root cells; thus, such signalling by K^+^ loss will have no major implications for overall K^+^ nutrition.

Box 2. **A model for cytosolic [K^+^] signalling**

The thickness of lines reflects transporter activity, and the relative size of each icon reflects the transporter’s expression level. Three phases are depicted. In the homeostatic phase (before stress) cytosolic [K^+^] is maintained at a constant level in both zones, but is slightly higher in the apex, due to both higher metabolic demand for K^+^ and 10–15 mV less negative membrane potential (MP) in this zone (e.g. Shabala *et al.*, 2016*b*). The optimal cytosolic [K^+^] levels in the mature zone are maintained mostly by low-affinity K^+^ uptake mediated by AKT channels along the electrical gradient provided by H^+^-ATPase. In the root apex, where the MP is less negative, a small but constant K^+^ leak via GORK channels occurs (as measured in MIFE experiments – e.g. Chen *et al.*, 2005; Shabala *et al.*, 2016*b*) and needs to be compensated by the high-affinity K^+^ uptake mediated by HAK transporters. Upon onset of the stress (the signalling phase), MP in the apical zone is depolarized to very low levels and triggers a massive K^+^ efflux via GORK channels that is further exacerbated by the increase in GORK transcripts. This efflux partially restores MP, and switches the cell’s operation from metabolic to defensive mode. Mature root cells also lose some K^+^, although to a much lesser extent, giving initially more-negative MP values. The stress-induced activation of H^+^-ATPase allows plant cells in both zones to recover K^+^ loss (the recovery stage). Some K^+^ obtained by mature zone cells may also be redirected symplastically to the apex, to enable cells to regain optimal [K^+^].

**Figure:**
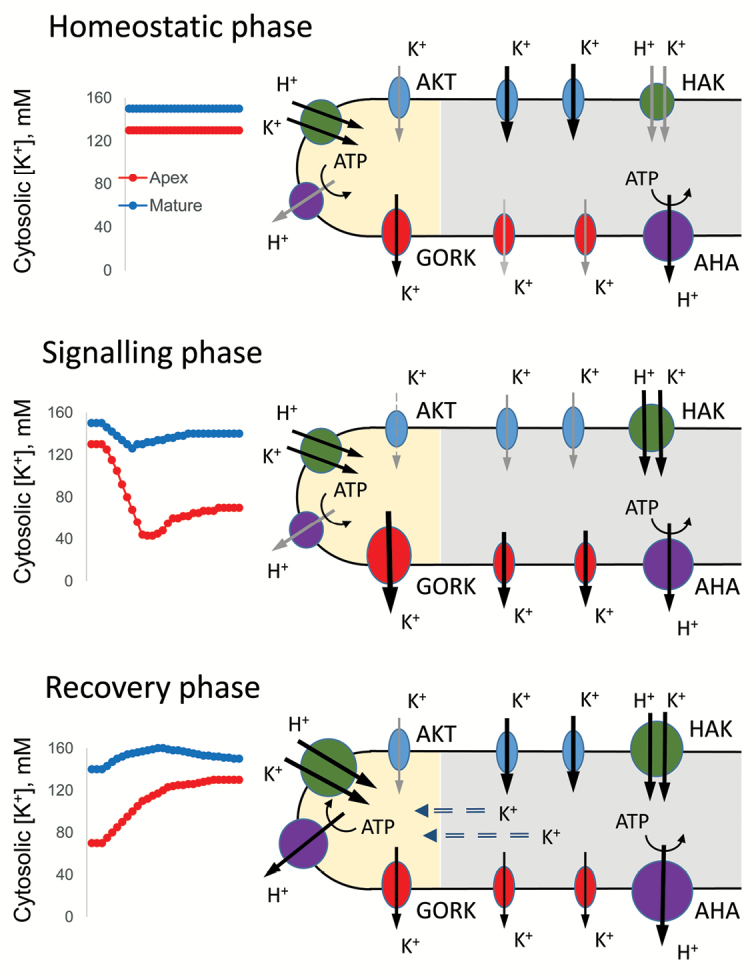

The timing of such signalling should be also considered. Given the connection noted above between potassium and PCD events, a prolonged decrease in the cytosolic K^+^ level may be detrimental to cell viability. Hence, signalling via K^+^ efflux should only be transient (Box 2) and kept under tight control. So, is it the right time to add transient cytosolic [K^+^] spikes to the list with Ca^2+^ and ROS, messengers that signal and shape plant adaptive stress responses?
